# Effect of Cryopreserved Amniotic Membrane Orientation on the Expression of Limbal Mesenchymal and Epithelial Stem Cell Markers in Prolonged Limbal Explant Cultures

**DOI:** 10.1371/journal.pone.0164408

**Published:** 2016-10-10

**Authors:** Zala Lužnik, Marko Hawlina, Elvira Maličev, Marina Bertolin, Andreja Nataša Kopitar, Alojz Ihan, Stefano Ferrari, Petra Schollmayer

**Affiliations:** 1 Eye Hospital, University Medical Centre, Ljubljana, Slovenia; 2 Blood Transfusion Centre of Slovenia, Ljubljana. Slovenia; 3 The Veneto Eye Bank Foundation (Fondazione Banca degli Occhi del Veneto), Zelarino-Venice, Italy; 4 Medical Faculty Ljubljana, Institute of Microbiology and Immunology, Ljubljana, Slovenia; Cedars-Sinai Medical Center, UNITED STATES

## Abstract

**Purpose:**

To evaluate the effect of prolonged limbal explants cultured without any scaffolds or on amniotic membrane (AM) on the viability, proliferation and differentiation potential of putative phenotypically defined cultured limbal mesenchymal (LMSC) and epithelial stem cells (LESC).

**Methods:**

Limbal explants were cultivated on cryopreserved intact AM or plastic plates using medium supplemented with only human serum. AM was positioned with either the epithelial or stromal side up. The outgrowing cells were immunophenotyped for the co-expression of mesenchymal stem cell markers (CD73/CD90/CD105 positive and CD45 negative), proliferation and putative progenitor markers (CXCR4, CD117), epithelial markers and antigen presenting cell markers (CD80, CD83, CD86) by flow cytometry. Immunohistochemistry on limbal cultures cultivated on AM was carried out with antibodies against pan-cytokeratin, p63, Ki67.

**Results:**

Morphological and immunostaining analyses revealed two distinct stem cell population types, which could be identified over prolonged culturing time periods. Expression of LMSC markers and CXCR4 was significantly higher (p < 0.05) in cultures cultivated without AM. However, no statistically significant difference was observed in CD117 expression. The cells cultivated on AM retained an epithelial cell structure, which was further confirmed by histology examination. Histology revealed limbal epithelial growth and p63, Ki67 positive cells on both sides of AM.

**Conclusion:**

Limbal cells cultivated on AM exhibited a lower expression profile of LMSC and CXCR4 markers as limbal cells cultivated on plastic culture plates. However, CD117 expression was similar. Histology confirmed limbal epithelial cell growth on both sides of AM, with no morphological differences, or positivity of cells for p63 and Ki67.

## Introduction

Corneal epithelium is renewed by stem cells (SC) located in the basal layer of the limbal epithelium (LE) in a special supporting microenvironment known as the limbal SC niche. The niche plays an important role in the maintenance of limbal epithelial SC (LESC) properties and is tightly regulated by factors from the surrounding tissue [1]. When the limbal SC containing niche is partially or totally damaged, a blinding and painful disease of limbal stem cell deficiency (LSCD) ensues [2].

Total and severe LSCD is difficult to manage. Transplantation of LESCs is necessary to restore vision [3,4]. In 1997, Pellegrini and colleagues first described transplantation of *ex vivo* expanded—cultured LE sheets containing LESCs (Cultivated Limbal Epihelial Transplanation) from a small amount of limbal tissue biopsy [5,6]. Since then, a variety of culturing techniques have been developed to optimise and standardise the *ex vivo* expansion of LE sheets on appropriate carrier substrates [6].

In a limbal explant culturing technique unprocessed limbal biopsy tissue can be cultured on a cryopreserved human amniotic membrane (AM) [3,7]. The AM serves both as an *ex vivo* surrogate limbal niche and as a carrier for successful LE expansion and transplantation. Galindo et al. already reported that cryopreserved intact human AM used as a culture carrier preserved stemness potential of cultured LESCs better than plastic culture plates alone [8]. Furthermore, intact AM enables limbal explant culturing without the need of a supportive 3T3 murine fibroblast feeder layer [9]. It is well known that intact AM consists of an epithelial monolayer with a thick basement membrane and an adjacent stroma—the spongy layer side, both exhibiting different biological properties [10]. The amniotic epithelium produces different growth factors, which may promote proliferation and differentiation of limbal epithelial cells [11]. Thus, limbal epithelial cells are preferentially cultured on the epithelial side of the AM (or on the basement membrane side if denuded AM is used). On the other hand, the AM stromal matrix has additional immunosuppressive function, which suppresses the expression of certain inflammatory cytokines that originate from the ocular surface epithelia [12], thus inhibiting fibrosis and myofibroblast differentiation [9].

As limbal explants are not enzymatically processed, the LESC are usually co-cultured with some of the underlying limbal stromal mesenchymal cells (LMC) [13]. Recently, small populations of limbal mesenchymal stem cells (LMSC) have also been observed in the anterior limbal stroma [14], with increasing evidence suggesting a direct role of LMSC in the provision of cells for corneal maintenance and regeneration [15]. Nevertheless, the importance of LMSCs for the LE *ex vivo* expansion and for the long-term success of LE transplant maintenance is still not well determined [1,13,15]. Moreover, different culturing conditions (e.g. culture media, carrier substrates [8]) can influence the phenotype and differentiation potential of cultured limbal epithelial and stromal mesenchymal SCs *in vitro*.

Therefore, these findings prompted us to examine the hypothesis that cryopreserved intact AM may influence not only LESC survival [8], but also the survival of limbal mesenchymal stromal and putative stem cells in limbal explant cultures. Secondly, the epithelial and stromal sides of the cryopreserved intact AM might have a different effect on distinct cultured cell populations.

The purpose of the present study is therefore to focus on the phenotypic characteristics of limbal mesenchymal as well as limbal epithelial SCs expanded from human cadaveric limbal explants cultured on cryopreserved intact AM or without it in a medium containing human serum as the only growth supplement that was already used in previous studies [7,16,17] using morphological and immunohistochemical techniques. As the *ex vivo* intrinsic biology of different limbal niche cells was intended to be studied, to avoid cellular damage or specific cellular phenotype selection [18], neither enzymatic nor other special surface treatment for explant adherence were used. The phenotypic limbal mesenchymal stem cell expression markers (the co-expression of CD73/CD90/CD105 positive and CD45 negative markers according to the International Society for Cellular Therapy (ISCT) criteria [19]), proliferation (Ki67) and differentiation potential (pan-cytokeratin) markers, the epithelial stemness/progenitor cell marker (p63) [8] and putative surface markers of LESCs [17] (CD117/c-kit and C-X-C chemokine receptor type 4 (CXCR4)) were being tested, as well as proliferation and activation status of antigen presenting cells (APC) in some primary limbal cultures (CD83, CD86, CD80).

Therefore, we herein report the first experimental study, which phenotypically demonstrated two distinct stem cell population types in limbal explant cultures cultivated on both sides of AM or without any scaffolds using a xenobiotic-free (animal-free) culturing model. Moreover, the long-term intrinsic proliferation dynamics of cultured putative LMSCs and LESCs are hereby reported.

## Materials and Methods

### Limbal explants harvesting

The local committee for Medical research Ethics of Slovenia approved all laboratory and tissue harvesting procedures (123/02/14, date: 25.3.2014). The research followed the tenets of the Declaration of Helsinki. Cadaveric human corneal tissues were collected after their next of kin gave written informed consent and only anonymized tissues were obtained from the Slovenian Eye Bank (Ljubljana, Slovenia). The corneoscleral tissues from 11 donors in the age range of 25–59 years (average age = 41.6 years, standard deviation [SD] = 10.9 years) were preserved for experiments after central corneal buttons were used for corneal transplantation purposes or from corneoscleral tissue, which did not meet the criteria for clinical use. These tissues were harvested within 12 hours after death and preserved in Optisol-GS (Bausch and Lomb Inc, Rochester, NY) at 4°C before use. The time from death to culture was 4.9 ± 3.4 days (range, 1–14 days).

### Limbal explant culturing

The limbal explant cultures were prepared using a modification of a previously described method [4]. In brief, the remaining limbal rim was cut into approximately 1 × 2 × 0.25 mm equal pieces of limbal explants (biopsy), which included the epithelium as well as some of the superficial limbal stromal tissue. Each limbal explant with the epithelium side down was directly placed into a well of twelve-well plastic culture plates (Falcon, Durham, North Carolina, USA) or on cryopreserved intact human AM (on the epithelial or stromal side), in a feeder cell-free culture system. Cryopreserved human AM were provided by the Blood Transfusion Centre of Ljubljana, Slovenia, and were preserved according to the method described by Koizumi et al [20]. Cryopreserved intact AMs were fastened onto a 35-mm culture plate with fibrin glue (Beriplast CSL Behring) with the epithelial or stromal side facing up for the flow cytometry studies. For histology and immunostaining experiments, the limbal explants were cultured epithelial side down on the AMs that were fastened in inter-lockable plastic rings, which were a generous gift from the Veneto Eye Bank Foundation (Venice, Italy).

The explants were cultured in a xenogenic-free culturing medium, which was an 1:1 mixture of Dulbecco modified Eagle medium (DMEM) (Gibco^™^/Thermo Fisher Scientific) and Ham's F12 medium (Gibco^™^/Thermo Fisher Scientific) containing 10% or 20% human serum (Sigma Aldrich, Italy) and antibiotics 50 μg ml^−1^ gentamicin and penicillin (Gibco^™^/Thermo Fisher Scientific), at 37°C under 5% CO_2_ and 95% humidity. The medium was renewed every 2–3 days. Growth was observed under an inverted microscope Zeiss Axiovert 100 (Carl Zeiss, Germany). The limbal explant cultures were incubated for 2–3 weeks until confluence was reached for the flow cytometry studies, when the cultures were harvested with 0.25% trypsin (Gibco^™^/Thermo Fisher Scientific) in 0.03% EDTA solution (Gibco^™^/Thermo Fisher Scientific) without the limbal explants. In the second set of experiments, to determine the long-term proliferation dynamics of cultured cells, limbal explant cultures were cultured over prolonged time periods. Prolonged limbal cultures were determined as limbal explant cultures cultivated over 30 days. To further extend the limbal explant cultures beyond the initial phase of primary culture (for 30–40 days), explants were repositioned epithelial side down to new culture plates for further cultivation (from primary to secondary culture) until confluence was reached. Therefore, the cumulative time period the limbal explants were cultured was 60–61 days (primary and secondary culture). Two limbal explants cultivated on plastic culture plates were cultivated for over 2 months (extended limbal explant cultures without passaging for 6 months).

### Cell viability assay

For the cellular death assay, the BD Pharmingen PE Annexin V Apoptosis Detection Kit I (BD Biosciences) was used. Each sample (n = 5) was washed in Binding Buffer 1x and centrifuged at 400 g during 5 minutes. After centrifugation, the supernatant was removed and the cells were re-suspended in 100 μL of Binding Buffer. Phosphatidylserine, which is a marker of early apoptosis, was stained with PE-(phycoerythrin) labelled Annexin V. Loss of membrane integrity as a consequence of necrosis and late apoptosis was detected by 7-aminoactinomycin D (7-AAD) staining of DNA. Data acquisition was performed with a FACS Calibur flow cytometer (BD Biosciences Immunocytometry Systems).

### Immunophenotyping of cultured limbal cells

The immunophenotype of limbal explant cell cultures was determined by flow cytometry. Monoclonal antibodies conjugated with fluorochromes were used for multiparameter flow cytometric analysis performed with BD FACS Aria or FACS Canto II cytometer (BD Biosciences Immunocytometry Systems), as was previously reported [21].

Limbal cells were characterized for the expression of markers: Anti-CK12 (FITC) (Santa Cruz Biotechnology; Germany), Anti-Mucin 5AC (MUC-5AC) (DyLight^®^ 650) (Abcam; UK)/ Anti-CK7 (FITC) (BD Pharmingen; San Jose, CA, USA); Anti-CD80 (APC) (Miltenyi Biotec; CA, USA) / Anti-HLA-DR (FITC) (BD Pharmingen; San Jose, CA, USA) / Anti-CD83 (PE) (Miltenyi Biotec; CA, USA) and Anti-CD86 (PerCP-Vio700) (Miltenyi Biotec; CA, USA); Anti-CD90 (FITC) / Anti-CD45 (PE) / Anti-CD105 (PerCP-Cy5.5) / Anti-CD73 (APC); Anti-CD184 (APC), Anti-CD117 (PE) (all from BD Pharmingen). Briefly, approximately 1x10^6^ limbal cells per ml were washed with PBS containing 1% BSA (both from Gibco^™^/Thermo Fisher Scientific) and incubated with the antibodies directed to the cell surface markers for 20 min at RT. In the case of intracellular staining, the cells were fixed and permeabilized with Invitrogen Fix and Perm reagents according to their protocol (Invitrogen, Austria), followed by incubation with primary antibody solution at 4°C for 30 min. After incubation, cells were washed with PBA containing 1% BSA and in the cases of non-conjugated Anti-Mucin 5AC antibody (Abcam; UK), the second staining was performed using a secondary antibody DyLight^®^ 650 goat anti-mouse IgG (H+L) (ab96879) (Abcam; UK) at 1/500 dilution and for the CK12 the secondary antibody FITC anti-mouse IgG (Santa Cruz Biotechnology; Germany) at the 1/100 dilution were used. Cells were incubated for 30 min at 22°C and washed twice with PBA containing 1% BSA before analyzation by flow cytometry. At least 3000 events were collected per sample. Cells were analyzed with BD FACS Diva software (BD Bioscience, San Diego, CA, USA). The FACS data are reported as mean fluorescence intensities (MFI) and as percentages of positive cells.

### Immunohistochemical staining of limbal explants on AM

Limbal explant cultures on AM were fixed in 4% paraformaldehyde. The fixed limbal explant outgrowths on AM were dehydrated in ascending alcohol series and embedded in paraffin, which were then cut into 4 μm thick sections with a microtome and mounted onto histological slides.

The limbal explant samples on AM were characterized for a pan-cytokeratin marker, a putative stemness marker p63, and proliferation marker Ki67. Monoclonal mouse anti-human p63 protein (Dako, Denmark), anti-pan-cytokeratin AE1/AE3 ((cytokeratin 1–19 detection), Novocastra) and anti-Ki67 (Dako, Denmark) were used. Expression of these markers in cultured limbal epithelial cells on AM was used with appropriate Dako visualization and control Kits (Dako, Denmark). Sample processing and data analysis were performed according to the manufacturer's instructions. Briefly, pre-treatment of formalin-fixed, paraffin-embedded tissue sections with heat-induced epitope retrieval (HIER) was performed using diluted EnVisionTM FLEX Target Retrieval Solution, High pH (50x). Deparaffinization, rehydration and epitope retrieval was performed in Dako PT Link, with pre-heat temperature of 65°C; epitope retrieval temperature and time was 97°C for 20 (±1) minutes; cool down to 65°C. Slide rack was removed from PT tank and immediately dip slided in jar/tank (e.g., PT Link Rinse Station) containing diluted room temperature EnVisionTM FLEX Wash Buffer (20x). Slides were left in Wash Buffer for 1–5 minutes. The recommended dilution of monoclonal antibodies and negative control reagent Dako Negative Control, Mouse IgG2a were used (Dako, Denmark). Positive and negative controls were run simultaneously with the limbal explant specimens. Two independent individuals carried out the quantification of positive cells using ImageJ software; multiple pictures were taken of each sample.

### Statistics

The results were expressed as the mean percentage of positive cells ± S.D./SEM. Two-tailed Student’s t-test was used to compare difference between two groups. A One-way analysis of variance (ANOVA) test was used to make comparisons among three or more groups. A p-value of < 0.05 was considered statistically significant. Each experiment was performed at least three times, if not otherwise stated.

## Results

**Fig 1:**
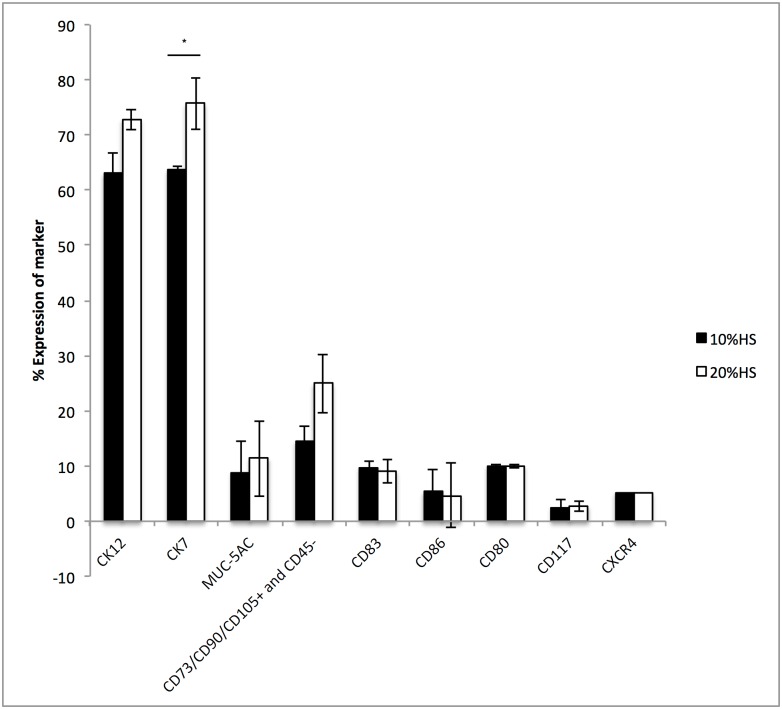
Results of flow cytometry profiles of confluent primary limbal explant cultures cultivated on plastic culture plates in only 10% or 20% human serum supplemented medium. A plot of the mean percentages of positive cells ± SEM for the tested markers in both tested HS concentration groups is shown (Student two-tailed t-test; * p < 0.05).

The initial culturing media validation experiments confirmed cellular outgrowth with a predominantly cobblestone-like epithelial appearance from primary limbal explants cultivated without AM on plastic culture plates in both tested media groups (10% and 20% HS supplemented media). The viability of cells cultured in both tested HS concentration groups was comparable (10% HS: 85.2 ± 1.8% and 20% HS: 80.7 ± 4.4%) with a similarly small percentage of apoptotic cells (10% HS: 7.1 ± 1.0% and 20% HS: 9.7 ± 2.7%) being found (t-test; p = 0.2). Next, the flow cytometry profile of cultured limbal cells was examined with a panel of monoclonal antibodies for epithelial (CK12, CK7, MUC-5AC), mesenchymal stem cell (CD73/CD90/CD105 positive and CD45 negative), putative surface marker fingerprints of LESCs (CD117/c-kit, CXCR4) and antigen presenting cell (APC) markers. Immunocytochemical analysis of confluent primary cultures is shown in Fig 1. The expression of CK7 was highly positive though lower in the cultures cultivated in 10% HS (63.7 ± 3.7%), when compared to cultures cultivated in 20% HS (75.7 ± 1.9%), the difference was statistically significant (t-test; p = 0.01). Interestingly, we found MUC-5AC positive cells in our cultures, a conjunctival goblet cell marker. As for CK7 marker, cells cultured in medium supplemented with 10% HS showed a lower expression of MUC-5AC (8.9 ± 0.6%) compared to cells cultured in 20% HS supplemented medium (11.4 ± 4.6%), however the difference was not statistically significant (t-test; p = 0.7). Therefore, to further elucidate the specific origin of cultured epithelial cells, the expression of cornea specific epithelial marker CK12 and the expression of CK7 marker were compared in a separate experiment. Again, higher expression rates for both markers were observed in 20% HS supplemented medium, with slightly higher percentage of CK7 positive cells (74.4 ± 2.2%) compared to CK12 positive cells (72.8 ± 5.5%), the difference was statistically not significant (t-test; p = 0.8). The opposite was observed in cultures cultivated in 10% HS supplemented medium (CK12: 63.1 ± 7.4% vs. CK7: 62.5 ± 5.3%; t-test: p = 0.9). Similarly, although a higher expression profile for mesenchymal stem cell markers (CD73/CD90/CD105 positive and CD45 negative) was expected in cultures cultivated in 20% HS supplemented medium (25.0 ± 6.9%) compared to 10% HS supplemented medium group (14.5 ± 5.7%), the difference was not statistically significant (t-test; p = 0.3). Moreover, the tested HS concentrations had only slight influence on the expression of putative LESC surface markers, the stemness marker CD117 (10% HS: 2.6 ± 0.3%; 20% HS: 2.7 ± 0.4%; t-test; p = 0.8) and cell migratory marker CXCR4 (10% HS: 5.2 ± 1.3%; 20% HS: 5.1 ± 0.9%; t-test; p = 0.9) and did not influence the *ex vivo* activation (CD83 positive cells) or proliferation of APC (the same percentage of cells in both tested groups; 10% HS: 10.1 ± 4.1%; 20% HS: 10.0 ± 5.8% of CD80 positive cells; t-test; p = 0.99). Therefore, as no statistically significant difference was observed in the expression profiles for mesenchymal and limbal epithelial stem cell markers, further experiments were continued with only 10% HS supplemented medium.

**Fig 2:**
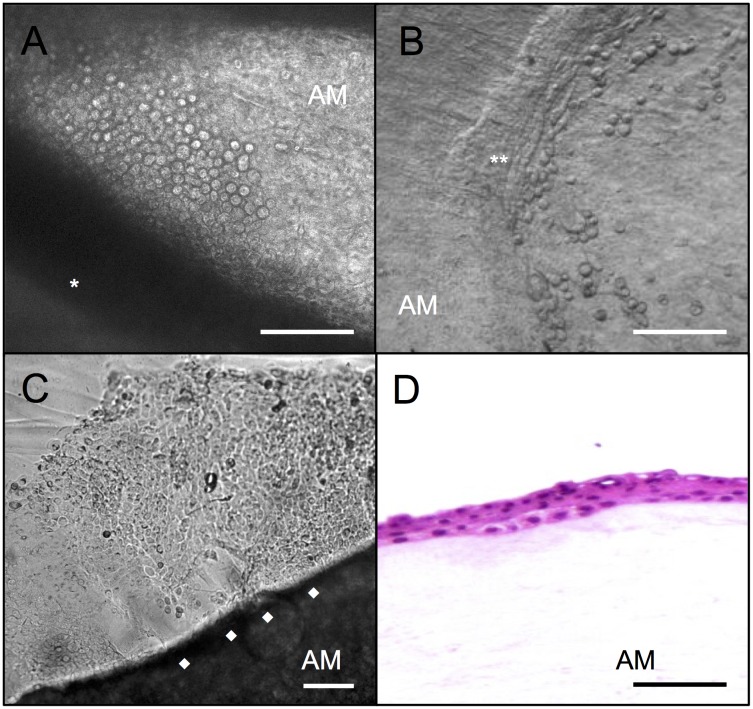
Limbal explant outgrowth of cells cultured on amniotic membrane after 3 days of culture and after 3 weeks of culture. The cells adjacent to the limbal explants were more uniform, smaller and had larger nuclei (A). In the first week of culture an epithelial line (B) was observed on AM (**), which after 3 weeks of culture reached the AM border (C) (◆) (A,B: Magnification:10x; C: Magnification:4x). Light microscopy showed limbal epithelial cells cultivated on AM (stromal side) produced a well-stratified cell layer (D); (HE: 10x). (*; explant; AM: amniotic membrane; HE: hematoxylin and eosin). Bars, 100 μm.

**Fig 3:**
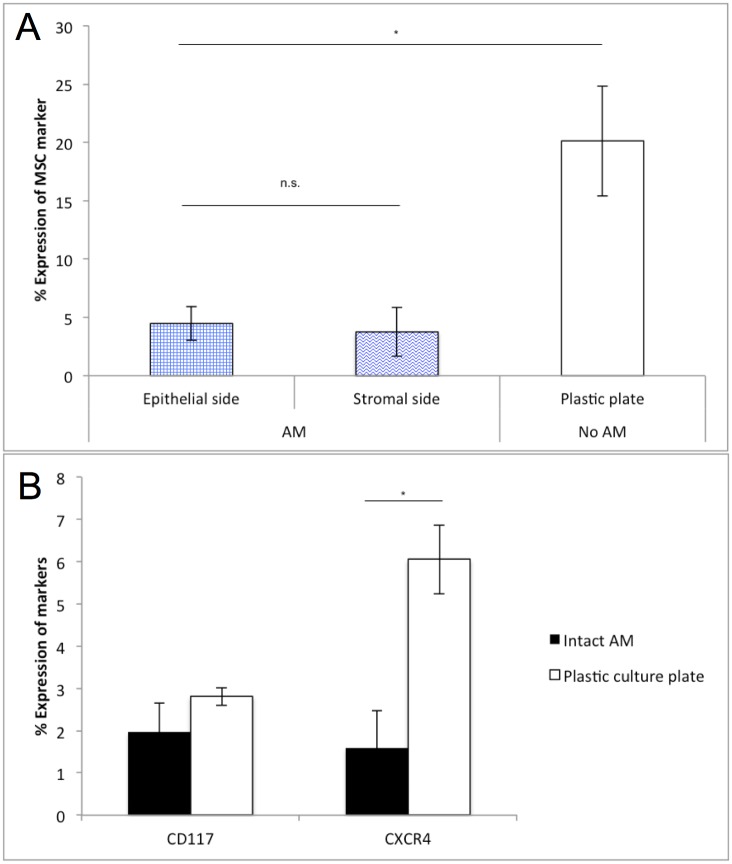
Expression of MSC markers (CD73/CD90/CD105^+^ and CD45^-^) and putative LESC surface markers (CD117 and CXCR4) in limbal explant cultures grown on both sides of amniotic membrane versus cultures cultivated on plastic culture plates. (A,B) The plots of the mean percentages of positive cells ± SEM for the tested markers are shown (A: ANOVA; B: Student two-tailed t-test; * p < 0.05; n. s. = not statistically significant). (A) On the epithelial side of the AM 4.5 ± 1.4% (n = 4) were positive and on the stromal side of the AM 3.8 ± 2.1% (n = 6) cells were positive for the mesenchymal stem cell marker. A statistically significant higher expression rate was found in primary limbal explant cultures (n = 13; 20.1 ± 4.7%) cultivated on plastic culture plates (ANOVA; p = 0.04). (B) CD117 was similarly expressed in limbal cell cultures cultivated on AM (n = 3; 2.0 ± 0.7%) compared to cultures cultivated on plastic culture plates (n = 3; 2.8 ± 0.2%; t-test; p = 0.2), CXCR4 expression was however significantly higher in cultures cultivated without AM (6.1 ± 0.8% vs. 1.6 ± 0.9%; t-test; p = 0.03). (MSC: mesenchymal stem cell; LESC: limbal epithelial stem cells.)

To further determine whether AM would influence the expression of mesenchymal stem cell and putative LESC surface markers on cultured limbal cells, limbal explants were cultured in parallel on epithelial (n = 4) or stromal side (n = 6) of the intact AM and on plastic culture plates (control group; n = 13). In all tested conditions, small and round cells with a scarce cytoplasm started to grow out from the attached explants by the 3^rd^ culturing day (Fig 2A). A clear leading epithelial edge (Fig 2B) was observed on both sides of the AM, which was covered within approximately 3 weeks (Fig 2C), when the cells were harvested for flow cytometry studies. Confluence in cultures cultivated without AM was reached in approximately 2 weeks. Primary limbal explant cultures on intact AM (on both sides) showed a statistically significant lower expression of mesenchymal stem cell markers (CD73/CD90/CD105 positive and CD45 negative) compared to primary limbal explants cultured without AM on plastic culture plates (ANOVA, p = 0.04) (Fig 3A). The expression of putative LESC surface markers was similar on both sides of AM. However, although CD117 was similarly expressed in limbal cell cultures cultivated on AM compared to cultures cultivated on plastic culture plates (t-test; p = 0.2), CXCR4 expression was significantly higher in cultures cultivated without AM (t-test; p = 0.03) (Fig 3B).

**Fig 4:**
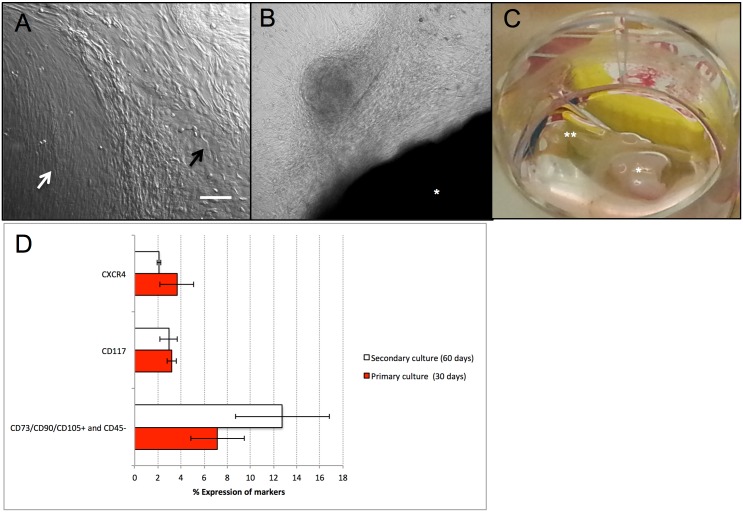
Morphological and flow cytometry profile results of prolonged limbal explant cultures cultivated without amniotic membrane. Limbal explant outgrowth of cells cultured on plastic culture plates in the absence of feeder layer cells and in medium supplemented with only human serum showed after 3 weeks of culture two morphologically distinct cell population types, a predominantly epithelial-like (black arrow) and a more fibroblast-like cell population (white arrow) (A). A sphere-shaped formation with spindle shaped cells around it was observed (B). (A,B: Magnification:4x). A limbal explant culture after 2 months of culturing without any passaging showing a translucent corneal stromal-like 3D tissue (C) (the total time of observation in culture was 6 months). A plot representing the mean percentages ± SEM (n = 7, paired two-sample t-test) of flow cytometry profiles, red bars representing the primary limbal cultures and white bars the transfered secondary limbal cultures, the differences not being statistically significant (p > 0.05) (D). (*) means limbal explant; (**) means translucent tissue. Bar, 100 μm.

To determine the long-term proliferation dynamics of cultured limbal mesenchymal and epithelial stem cells, limbal explant cultures were cultured over prolonged time periods (cumulatively for 60 days). Thus, to further extend the limbal explant cultures (cultured without AM) beyond the initial phase of 30 days, at which time the outgrowths expressed two morphologically distinct cell population types (Fig 4A), explants were repositioned epithelial side down to new culture plates without AM (n = 7) for additional 30 days. Flow cytometry studies after each cycle showed a 2-fold increase in the mesenchymal stem cell marker expression population found in the secondary cultures (primary culture 7.2 ± 2.3%; secondary culture 12.8 ± 4.1%; paired two-sample t-test; p = 0.3), however the percentages of CD117 positive (primary culture 3.2 ± 0.4%; secondary culture 2.9 ± 0.8%; paired two-sample t-test; p = 0.8) and CXCR4 positive (primary culture 3.6 ± 1.5%; secondary culture 2.1 ± 0.2%; paired two-sample t-test; p = 0.4) cells remained comparable between primary and secondary cultures (Fig 4D). Additionally, we observed sphere-shaped formations with spindle shaped mesenchymal-like cells around them (Fig 4B) and after 2 months of culture a macroscopically visible, translucent 3D tissue was formed (Fig 4C).

**Fig 5:**
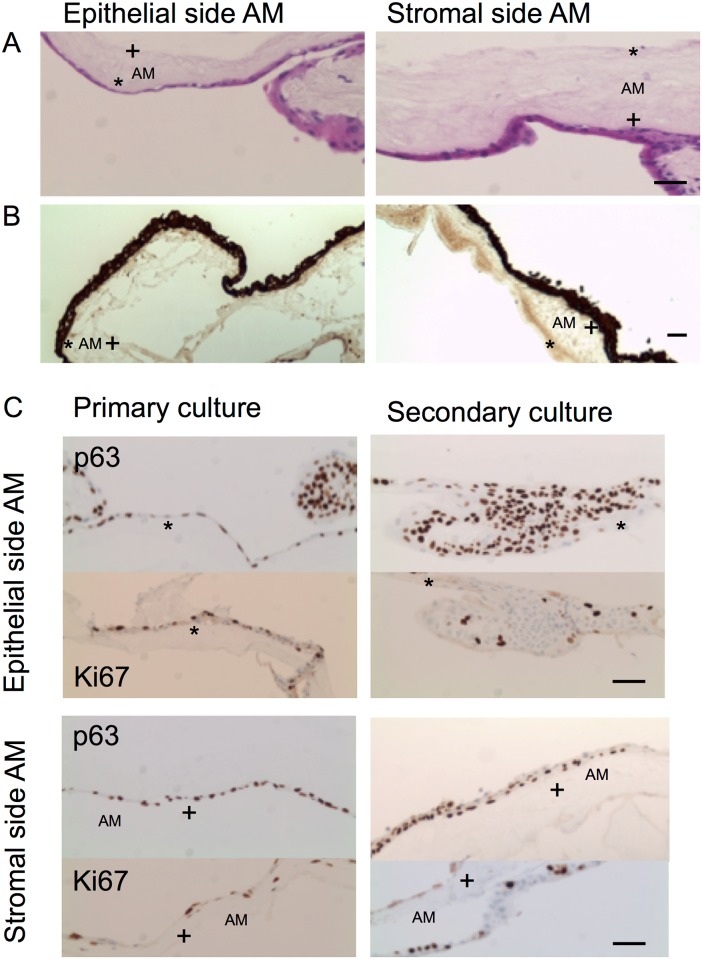
Immunohistochemical comparison of primary and secondary limbal explant cultures cultivated on the epithelial or stromal side of the AM in a human serum supplemented medium. In primary limbal explant cultures cultivated for 14 days, limbal epithelial growth was observed on both sides of the AM on HE sections (A), similarmorphologywas observed in secondary limbal explant cultures on both sides of the AM after the limbal explants were cultured for 40 days on plastic culture plates and stained positive for pan-cytokeratinmarker after 21 days of culture on AM (B). Cells from primary and secondary cultures on both sides of the AM (explants transferred after 14 days, from the same donor) expressed p63 and Ki67 marker, however in the secondary cultures a slight decrease was observed for Ki67 marker expression (C). (Magnification A:20x, B:10x; C:20x). (AM: amnioticmembrane; epithelial side, + stromal side; HE: hematoxylin and eosin). Bars, 50 μm.

In a separate experiment we compared primary limbal explant cultures cultured on both sides of the AM (n = 6) with secondary limbal explant cultures cultured on both sides of the AM for additional 21 days after primary extended culturing (for 40 days) on plastic culture plates (n = 6). Under both culturing conditions, cells started to migrate from the limbal edges to form an epithelial sheet. Light microscopy of hematoxylin and eosin stained sections of the *ex vivo*-generated limbal epithelial sheets revealed a one to two-layered epithelium with basal column-shaped cells and superficial flattened squamous-like cells, intermittently a stratified roughened epithelium was noticed resembling limbal epithelial structure (Fig 5A). Immunohistochemistry showed uniform distribution of pan-cytokeratin marker in all epithelial layers (Fig 5B). The putative stem cell marker p63 and proliferation marker Ki67 were expressed in basal cells cultured on both side of the AM, indicating active proliferation in primary and secondary cultures. Next, two limbal explants from the primary cultures on AM were, after 14 days of culturing, transferred into new cultures on AM for another 14 days, before histology was done. Immunohistochemistry showed again similar expression of p63 and Ki67 markers (Fig 5C).

## Discussion

Viable cell outgrowths of putative LESCs and LMSCs from human limbal explants cultured on plastic plates without any scaffolds or on the epithelial or stromal side of the AM could be characterized morphologically and immunophenotypically over prolonged culturing time periods in a xenogenic-free selective medium. The comparative results of this study clearly present additional experimental evidence that AM scaffolds, irrespective of the orientation, preferentially preserved and expanded LE and LESCs even after prolonged culturing, serving as a protective limbal niche. However, in prolonged limbal explant cultures cultivated on plastic plates without any scaffolds, the LMC and putative SC population, which was determined with the simultaneous co-expression of ISCT determined positive markers CD73/CD90/CD105 and a negative marker CD45 [19], expanded giving the impression of an intrinsic feeder layer cell population, which might support long-term survival and protection of cultured LE cells. As most of the translational experimental studies use short-term limbal explant cultures on different scaffolds, the LMSC population and their important supportive functions are not usually taken in consideration. Thus, the purpose of this recent study was to further investigate the influence and importance of LMCs and putative SCs in limbal explant cultures.

Morphologically, prolonged limbal explant cultures on plastic culture plates clearly demonstrated two distinct cell population types in our preliminary experiments, the observations being in consistence with previous reports [1,14,15,22]. However, current media that are used for limbal epithelial culture are reported to be suboptimal for mesenchymal stem-like cell culture [15]. Culturing medium supplemented with only 10% HS is already in use for short and long-term *ex vivo* LE culturing [7,16,17]. On the other hand, higher serum concentration rates might enable better survival of mesenchymal stem-like cells in culture [15]. Hashmani et al. [15] reported that the 20% fetal bovine serum supplemented M199 medium was superior for LMSC culture compared to standard DMEM based medium supplemented with 10% fetal bovine serum [15]. Therefore, as 10% HS supplemented medium might not enable proper survival of both limbal epithelial and mesenchymal stem-like cells in culture, we decided first to compare the phenotypic potential of cells cultured in either only 10% HS or 20% HS supplemented culturing medium.

As expected, our flow cytometric studies of the primary cell cultures cultivated without a scaffold confirmed two distinct cell lineages in both tested culturing media groups. The epithelial cell phenotype was confirmed with positivity for cytokeratin 7, a marker that is highly expressed in limbal and conjunctival epithelium and is lost during the differentiation process into corneal surface epithelium [23], and with a terminally differentiated cornea-specific cytokeratin marker CK12. Both cytokeratin markers were similarly expressed and the predominant cell types observed in our study (63.1–72.8% CK12 and 62.5–75.7% CK7 positive cells). Jirsova et al. reported that high positivity for CK7 (around 100%) first appeared in the superficial epithelial layers of the limbal area, with the central corneal epithelium being completely negative for CK7 [23]. Therefore, the results might indicate that in our study a mixture of different ocular epithelial cells has been obtained from the limbal explants, a combination of mature corneal epithelial cells (CK12 positive), limbal (CK7 positive) and also conjunctival epithelial cells (CK7 positive), especially as some cells were additionally positive for MUC-5AC marker, a conjunctival goblet cell marker [24]. As limbal/corneal and conjunctival epithelial lineages are closely related (having developed from the same set of pre-ocular PAX6-positive ectodermal cells) [24], we could speculate, that the high serum concentrations supplemented to our culturing medium might also provide sufficient supporting growth factors for cells to differentiate into goblet-like cells (e.g. from the cells in the border of limbal explants). The reverse process of cultured conjunctival epithelium trans-differentiation into corneal epithelium has already been reported in a rabbit eye surface model, as after only two weeks the transplanted cultured conjunctival epithelial sheets on the *in vivo* corneal stromal environment lost the expression of MUC-5AC marker and started to express CK3 and CK12 markers [24]. However, additional experimental studies need to elucidate these findings, especially as conjunctival/limbal epithelium co-cultivation and transplantation is recently being studied for clinical application [25] to treat severe dry ocular surface disorders with additional conjunctival epithelium damage and goblet cell loss.

The second cell type exhibited a spindle shaped, fibroblast-like appearance similar to that reported by Polisetti et al. [1]. These cells demonstrated the ability to form round sphere-shaped formations, giving the impression of embryoid bodies [1], which might have mesenchymal stem cell potentials. Therefore, we tested the cultures for the expression of ISCT defined phenotypical LMSC markers [19].

Limbal MSCs were reported to be typically CD34, CD45 and HLA-DR negative and CD73, CD90, and CD105 positive [13, 15], however, to our knowledge, no previous study reported on the LMSC population in limbal explant cultures using flow cytometric studies and identification methods for simultaneously co-expression of defined markers (cells simultaneously positive for CD73/CD90/CD105 and negative for CD45 marker as suggested by ISCT [19]). Nevertheless, it was recently reported that a sub-population of limbal stromal cells located beneath the limbal crypt rich regions were highly positive for either CD90 or CD105 MSC markers [14]. Herein, we found from 7.2% to 24.5% of cells that were positive for mesenchymal stem cell marker expression (simultaneously CD73/CD90/CD105 positive and CD45 negative), and additionally expressed principal criteria for the identification of mesenchymal-like stem cells, as are fibroblast-like morphology and adherence to plastic [26]. Zakaria et al. reported that around 80% of cultured cells were positive for only CD73 marker and fewer than 10% of cells were positive for CD90 marker in primary limbal explant cultures cultivated in a xenogenic-free culturing medium [27], however the results cannot be directly compared. To date, Hashmani et al. already demonstrated that cultured limbal stromal and peripheral corneal stromal cells produced a mesenchymal stem cell population [15], which complied with all the defined ISCT MSC criteria [19]. However, in the current study the functionally based property for trilineage mesenchymal differentiation of the putative LMSC population was not tested. Therefore, the identified limbal mesenchymal cell population conformed only partially to the ISCT guidelines for MSC characterization [19], which is a major limitation of our current study.

Although a slightly higher percentage of LMSC marker positive cells was observed in cultures cultivated in 20% HS supplemented medium, the difference was statistically not significant. Furthermore, irrespective of the HS concentration used in medium, we observed around 2–3% of cells, which were positive for CD117, a cytokine receptor expressed on the surface of different stem cells, and around 7% positive cells for CXCR4 (CD184), a chemokine receptor for proliferating cells. Both markers have been recently reported as putative LESC surface markers [17]. These results may suggest that in our primary limbal explant cultures around 2–7% of cells were LESCs. These results can be compared with the recent study by Szabo et al., which found around 0.6% of CD117 positive cells and around 22% of CXCR4 positive cells in primary limbal explant cultures [17]. However, future functional test are needed to further specify the correct origin and stemness potential of these cells.

Importantly, after consecutive prolonged limbal explants culturing on plastic culture plates the percentage of putative LMSC marker positive cells increased, whereas the expression of CD117 and CXCR4 slightly decreased or remained stable. This might indicate the important supportive role of the putative LMSCs for the LESC maintenance in prolonged *ex vivo* cultures. These findings are consistent with the publication of Ainscough et al., which demonstrated that LMC cultures stimulated limbal epithelial cell growth *in vitro* and could function as intrinsic feeder cell layer [22]. Although the expression of CD117 and CXCR4 markers was reported to be lost in long-term limbal epithelial cultures [17], the LMSC population was not determined in this study and could be a reason for the difference. Similarly, we have observed a transparent 3D tissue resembling corneal tissue after several months of cultivation, which may further indicate the important interactions between putative LMSCs and LESCs.

In contrary, if the limbal explants were cultivated on cryopreserved intact AM, the expression of LMSC markers significantly decreased, with around 4% of LMSC marker positive cells being found in cultures cultivated on the epithelial or stromal side of the AM. Although CD117 was similarly expressed in limbal cell cultures cultivated on intact AM compared to cultures cultivated on plastic culture plates, CXCR4 expression was significantly lower in cultures cultivated on AM, indicating that the migratory stimulus was down regulated in confluent limbal cultures on AM. Thus, the flow cytometry results confirmed that AM scaffolds preferentially support the proliferation of limbal epithelial cells.

Furthermore, histology sections of secondary limbal explant cultures on AM (after prolonged primary culture on plastic culture plates to propagate limbal mesenchymal proliferation) showed a basal cell layer of limbal epithelial cells that were uniformly small and cuboidal, and had a scanty cytoplasm. The epithelium was positive for pan-cytokeratin antibody staining, a marker that recognizes different keratins in simple and stratified epithelium, as previously reported by Li et al. [4]. We observed a uniform distribution of p63 positive small cells in the basal cell layers over the AM. The expression of p63 transcription factor is well known to be located in the basal layers of the limbal epithelium, restricted to cells with high proliferative potential [28] such as limbal epithelial stem and progenitor cells (e.g. transitory amplifying cells) [4, 28]. Di Iorio and colleagues [29] have shown the correlation between cell size and the expression intensity of p63 marker and the more limbal epithelial specific stem cell isoform ΔNp63alpha (cell diameter of 10 μm). Of note, as in the recent study the ΔNp63alpha isoform was not used, the detected p63 positive cells with larger diameters than 10 μm might represent also the transitory amplifying cell population [29, 30].

Furthermore, some basal cells were found positive for Ki67, a proliferation marker. At the border of the limbal explant the outgrowing epithelium showed stratification similar to limbal epithelium.

Although some previous studies reported a lack of cell outgrowth from limbal explants that were cultured on AMs with thick, intact stromal layer [31], we have observed cell outgrowth on both sides, with similar proliferation rates and morphology in primary and secondary cultures. Our limbal explant cultures were cultured submerged in medium with higher human serum concentrations, which might partially explain the difference. The limbal biopsies from the reported studies were cultured at the air-liquid interface until an outgrowth of approximately 6 mm in diameter was observed, thus the thick spongy layer might have interfered with nutrient diffusion, as the authors have explained [29]. In our secondary cultures the previous *ex vivo* culturing on plastic culture plates might have also additionally stimulated the wound healing processes in the limbal explants, promoting better cell growth. However, this might not be the case in our primary limbal explant cultures on both sides of the AM where a similar epithelial stratification morphology was observed on the epithelial and stromal side of the AM, with similar expression of p63 and Ki67 positive cells.

These data indicate that the epithelial progenitor cells cultured from limbal explants are indeed preserved during prolonged culturing (primary and secondary limbal explant cultures on AM showed similar immunohistochemical profiles) and that the AM enables epithelial formation after long-term culture. Furthermore, we did not observe cell outgrowth from secondary central corneal explant (n = 1) on AM, and only slight outgrowth from secondary peripheral corneal culture (n = 1), which is in consistence with previous studies [3]. These observations might further indicate, that AM cultures are more selective for supporting the growth of limbal epithelial progenitor cells [3].

Moreover, a previous study by Li et al. suggested that during serial cultivation, the epithelial cells invaded into the limbal explant stroma undergoing epithelium-mesenchymal transition [4]. The intrastromal invasion of epithelial cells happened in limbal explants cultured epithelial side up in the submerged culture systems [4]. Of note, we cultured the limbal explants epithelial side down, so the limbal epithelium was not in direct contact with the medium. Nevertheless, in the present study, we could also observe intrastromal epithelioid cell invasion (pan-cytokeratin positive) in two histological samples, however further studies should be undertaken to determine the fate of these cells.

In summary, to our knowledge, this is the first experimental study, which morphologically and immunophenotypically simultaneously identified two putative distinct stem cell population types in prolonged limbal explant cultures in a xenogenic-free selective medium. In limbal explant cultures cultivated on AM fewer LMSC marker positive cells were observed, with immunohistology revealing limbal epithelial proliferation and p63 and Ki67 expression on the epithelial and stromal side of the AM, even after serial cultivation of limbal explants. Thus, AM scaffolds have preferentially preserved and expanded limbal epithelial cells. However, in prolonged limbal explant cultures cultivated without any scaffolds, the LMC and putative SC population expanded giving the impression of an intrinsic feeder layer cell population, which might support long-term survival and protection of cultured LE cells, presuming also a possibility for use in tissue engineering as a source of autologous feeder layer cells.
